# An explorative single-arm clinical study to assess craving in patients with alcohol use disorder using Virtual Reality exposure (CRAVE)—study protocol

**DOI:** 10.1186/s12888-023-05346-y

**Published:** 2023-11-14

**Authors:** A. Lütt, N. Tsamitros, T. Wolbers, A. Rosenthal, A. L. Bröcker, R. Schöneck, F. Bermpohl, A. Heinz, A. Beck, S. Gutwinski

**Affiliations:** 1 Psychiatric University Hospital Charité at St. Hedwig Hospital, 10115 Berlin, Germany; 2 Department of Psychiatry and Psychotherapy, Charité - Universitätsmedizin Berlin, Corporate Member of Freie Universität Berlin, Humboldt-Universität Zu Berlin, Campus Charité Mitte, 10117 Berlin, Germany; 3 Berlin Institute of Health at Charité, Universitätsmedizin Berlin, 10117 Berlin, Germany; 4 German Center for Mental Health (DZPG), partner site Berlin, Berlin, Germany; 5 German Center for Neurodegenerative Diseases (DZNE), 39120 Magdeburg, Germany; 6 Salus Clinic Lindow, 16835 Lindow, Germany; 7 Faculty of Health, Health and Medical University, 14471 Potsdam, Germany

**Keywords:** Virtual Reality, Alcohol Use Disorder, Cue Exposure Therapy, Craving, Psychophysiological parameters, Heart rate variability, Electrodermal activity, Pupillometry

## Abstract

**Background:**

Alcohol use disorder (AUD) belongs to the most burdensome clinical disorders worldwide. Current treatment approaches yield unsatisfactory long-term effects with relapse rates up to 85%. Craving for alcohol is a major predictor for relapse and can be intentionally induced via cue exposure in real life as well as in Virtual Reality (VR). The induction and habituation of craving via conditioned cues as well as extinction learning is used in Cue Exposure Therapy (CET), a long-known but rarely used strategy in Cognitive Behavioral Therapy (CBT) of AUD. VR scenarios with alcohol related cues offer several advantages over real life scenarios and are within the focus of current efforts to develop new treatment options. As a first step, we aim to analyze if the VR scenarios elicit a transient change in craving levels and if this is measurable via subjective and psychophysiological parameters.

**Methods:**

A single-arm clinical study will be conducted including *n* = 60 patients with AUD. Data on severity of AUD and craving, comorbidities, demographics, side effects and the feeling of presence in VR will be assessed. Patients will use a head-mounted display (HMD) to immerse themselves into three different scenarios (neutral vs. two target situations: a living room and a bar) while heart rate, heart rate variability, pupillometry and electrodermal activity will be measured continuously. Subjective craving levels will be assessed before, during and after the VR session.

**Discussion:**

Results of this study will yield insight into the induction of alcohol craving in VR cue exposure paradigms and its measurement via subjective and psychophysiological parameters. This might be an important step in the development of innovative therapeutic approaches in the treatment of patients with AUD.

**Trial registration:**

This study was approved by the Charité—Universitätsmedizin Berlin Institutional Review Board (EA1/190/22, 23.05.2023). It was registered on ClinicalTrials.gov (NCT05861843).

**Supplementary Information:**

The online version contains supplementary material available at 10.1186/s12888-023-05346-y.

## Background

Alcohol use disorder (AUD) is a severe disorder leading to a substantial burden of disease with worldwide 3 million deaths per year [1]. Current treatment approaches yield unsatisfactory long-term effects with relapse rates up to 85% [2]. Both the individual and their environment are strongly affected by the disorder. Including all cost components (direct and indirect cost estimates of e.g., health care, unemployment or premature mortality) associated to harmful alcohol use, the costs would equal about 2,6% of the Gross Domestic Product (GDP) in the examined countries [3]. Alcohol craving as the strong desire to drink, is a major predictor for relapse and a main diagnostic criterion [4, 5]. Craving is associated with psychological and physiological responses [6] and can be intentionally induced by confronting patients with alcohol-related cues according to the cue-reactivity paradigm [7], such as a bar or a glass of wine.

The intentional presentation of such conditioned, contextualized cues is used as part of „Cue Exposure Therapy “ (CET) in Cognitive Behavioral Therapy (CBT), aiming to enable patients to identify and handle individual relapse risks [8]. Cue Exposure Therapy has shown substantial clinical effects, although recent meta-analyses emphasized the small number and questionable quality of existing trials [9, 10]. Although it is an effective strategy, CET for patients with AUD has not yet been established in clinical routine, because of the high organizational, timely and financial costs [8]: a) actors need to be available, b) to assure realistic settings, real bars have to be visited or laboratory-based bars have to be created and c) the change between different contexts is limited [11]. Virtual Reality (VR) is a new technology that is currently developing rapidly across several fields, and VR-based therapies are about to become a major component of digital mental health [12, 13]. Using e.g., head-mounted displays (HMD) patients can immerse into a computer-generated 3D world, where realistic spatial and social interactions are possible in real-time [14]. VR interventions can be used for both therapeutic and diagnostic approaches and have been established for various psychiatric indications such as anxiety disorders (e.g., specific phobias, social anxiety disorder) and post-traumatic stress disorder. [13, 15]. Innovative approaches including virtual avatars in the therapy of psychotic disorders show promising results [12]. Furthermore, VR applications have received increasing attention in the field of substance use disorders (SUD) [16, 17]. VR yields several advantages compared to in vivo exposure: it enhances practicability of CET, allows comparability between treatment providers and offers high ecological validity by using multimodal dynamic stimuli in an immersive environment [15, 18].

The assessment of craving in VR paradigms can provide a basis for the development of an effective exposure therapy, allowing to understand which contextual cues elicit pronounced cue reactivity and should therefore be used for habituation and extinction learning in cue exposure therapy. Additionally, craving assessment can serve as an individually tailored diagnostic tool helping to identify high-risk situations and automatic responses. Studies on craving assessment in patients with AUD show that different alcohol-associated VR-scenarios successfully induce craving [17]. Interestingly, a recent study showed that craving was significantly related to the sense of presence, meaning the perceived ecological validity of the virtual environment, underlining the importance of a realistic, high-end 3D VR environment [19]. However, apart from one study using electroencephalography (EEG, [20]) only subjective craving parameters (e.g., visual analogue scales, VAS) were used [16]. VAS are the international standard but have several disadvantages: They rely on subjective reports and the assumption that patients with AUD can perceive and specify their own craving. VAS are also biased with respect to social desirability, meaning that individuals may not answer completely truthfully, but with respect to what is socially acceptable.

Psychophysiological parameters of craving (e.g., changes in electrodermal activity, pupil size and heart rate) are well established in studying physiological cue reactivity [6]. A recent meta-analysis confirmed the link between cue-induced craving and psychophysiological cue reactivity to alcohol use and relapse [7]. Considering specific physiological parameters, a review with 33 included studies confirmed the association between reactive HRV to alcohol cues and craving, faster relapse and negative mood [21]. High frequency HRV (HF-HRV), which reflects parasympathetic activity, has been shown to be increased by alcohol-related cues [22, 23]. Furthermore, a pilot study assessing pupil reactivity to alcohol-related and neutral cues as a predictor for relapse, showed that alcohol-dependent patients reacting with greater pupillary dilation were more prone to relapse at a 4-month follow-up [24].

Apart from one older study which examined electroencephalographic correlates of craving in a small cohort of 20 patients [20], physiological parameters have not been used as outcomes for VR-exposure in patients with AUD, yet [16]. In VR-studies on methamphetamine use disorder physiological data (heart rate variability, eye tracking, electrodermal activity) were able to discriminate between patients and healthy controls [25, 26].

The planned study we present here, aims to assess if VR exposure can induce craving, being intentionally induced by different VR scenarios, with help of subjective and objective parameters.

### Primary hypothesis

We hypothesize that exposure to alcohol-related scenarios in VR will elicit a transient increase in craving in patients with AUD, measurable with subjective and physiological parameters compared to exposure in a neutral VR environment.

### Exploratory objectives:

- To identify VR-contextual cues predictive of pronounced craving
- To assess craving duration over the first 3 h following the exposure
- To examine the influence of the sense of presence in VR on craving levels
- To assess motion sickness and its correlation to craving

## Methods and analysis

### Study design

This is a prospective, explorative, single-arm, study that will be conducted in the Psychiatric University Hospital Charité at St. Hedwig-Hospital, Berlin. Second participating recruitment site is the Salus Clinic Lindow. The study is approved by the Ethic committee of the Charité—Universitätsmedizin Berlin (EA1/190/22).

### Recruitment

Patients with AUD treated in the inpatient or outpatient psychiatric clinics of the Psychiatric University Hospital Charité at St. Hedwig-Hospital or Salus Clinic Lindow will be contacted by the study personal. If interested, they receive the study information and will be screened for eligibility during a short interview.

### Inclusion criteria

- age: 18–65 years
- diagnosis of alcohol dependence according to ICD-10 (F10.2)
- completed in-patient withdrawal treatment during the last 3 months
- history of alcohol craving, confirmed via craving questionnaires
- able to provide written informed consent

### Exclusion criteria:

- substance dependence other than alcohol and nicotine
- current alcohol intoxication (randomly tested via measurement of breath alcohol concentration)
- unable to understand the study information, consent form or principles of the study
- abstinence for less than 7 days or on-going consumption of alcohol
- severe neuropsychiatric disorder, e.g., schizophrenia spectrum disorders, bipolar affective disorder or substantial cognitive impairment
- serious illnesses influencing brain-/heart-function with influence on physiological study parameters.
- acute suicidality (or acute endangerment of others)
- concurrent pharmacological treatment targeting AUD (i.e. benzodiazepines) or craving (i.e. acamprosate, disulfiram, naltrexone, nalmefene) and further medication significantly influencing heart frequency.

### Data collection

#### Clinical data and questionnaires

**Fig. 1:**
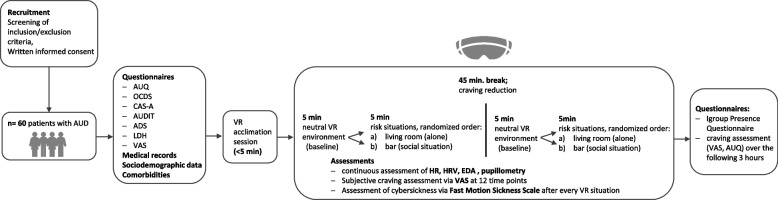
Workflow CRAVE-study. Abbreviations: AUQ – Alcohol Urge Questionnaire, OCDS – Obsessive Compulsive Drinking Scale, CAS-A – Craving Automated Scale for Alcohol, AUDIT – Alcohol Use Disorders Identification Test, ADS – Alcohol Dependence Scale, LDH – Lifetime Drinking History, VAS – Visual Analogue Scale, HR – heart rate, HRV – heart rate variability, EDA – electrodermal activity

General baseline data are collected in interviews before the VR exposure. These potentially confounding variables include demographic data (socioeconomic status), age, gender, medical history and comorbidities. Screening interviews include the Alcohol Urge Questionnaire (AUQ, [27]), Obsessive Compulsive Drinking Scale (OCDS, [28]) and Craving Automated Scale for Alcohol (CAS-A, [29]) to ensure the capability of perceiving and indicating subjective craving in patients. Severity of alcohol dependence will be measured with the Alcohol Use Disorder Identification Test (AUDIT, [30]), the Alcohol Dependence Scale (ADS, [31, 32]) and the Lifetime Drinking History (LDH, [33]). VAS scores (scores from 0–100)—the international standard measurement of subjective craving [34]—will be assessed before, during (3 times during each VR environment, minutes 00:30, 02:30, 04:30, 05:30, 07:30, 09:30, 10:30, 12:30, 14:30, 15:30, 17:30, 19:30) and directly after the VR exposure (min. 20:30). Furthermore, we will assess VR-specific side effects (kinetosis) using the Fast Motion Sickness Scale [35] during VR exposure. To study the short-term effects of VR exposure on craving, participants will indicate their craving level every hour for 3 h after exposure on a VAS and via the AUQ. To evaluate the feeling of “presence” during VR exposure, participants will be asked to complete the Igroup Presence Questionnaire (IPQ, [36, 37]) after completing VR exposure. The assessment of clinical data and questionnaires is estimated to take approximately 90 min. (Fig. 1; Workflow).

#### Psychophysiological data

To analyze the effect of VR exposure on craving-related physiological markers, we will assess heart rate (HR), heart rate variability (HRV), electrodermal activity (EDA) and pupillometry. Measurements will take place continuously during VR exposure conditions.

In order to collect cardiovascular (HR, HRV) and EDA data, we will employ the BIOPAC MP 160 system (Biopac Systems, Santa Barbara, CA, USA) together with a BioNomadix- RSPEC and 3-lead contact electrodes as well as the BioNomadix PPGED and a wireless remote sensor. The eye-tracker used to capture event-related pupil dilation is integrated into the VR headset (VIVE Pro Eye).

We will employ different preprocessing and analysis methods to extract HRV parameters in the time- (e.g., standard deviation of all interbeat intervals) and frequency domain (e.g., high/low frequency HRV). Here, we will use the AcqKnowledge® software provided by Biopac Systems and perform automated analyses of parameters of interest according to international measurement standards [38]. Tonic EDA will be measured with skin conductance level extrapolated via continuous decomposition analysis (CDA) [39]. HRV as well as EDA parameters will be averaged across the conditions (pre, post and during different exposure scenarios). Pupil diameter and blink rate during exposure scenarios will be analysed using algorithms by SomaReality (Soma Reality GmbH) [40].

#### VR exposure

**Fig. 2:**
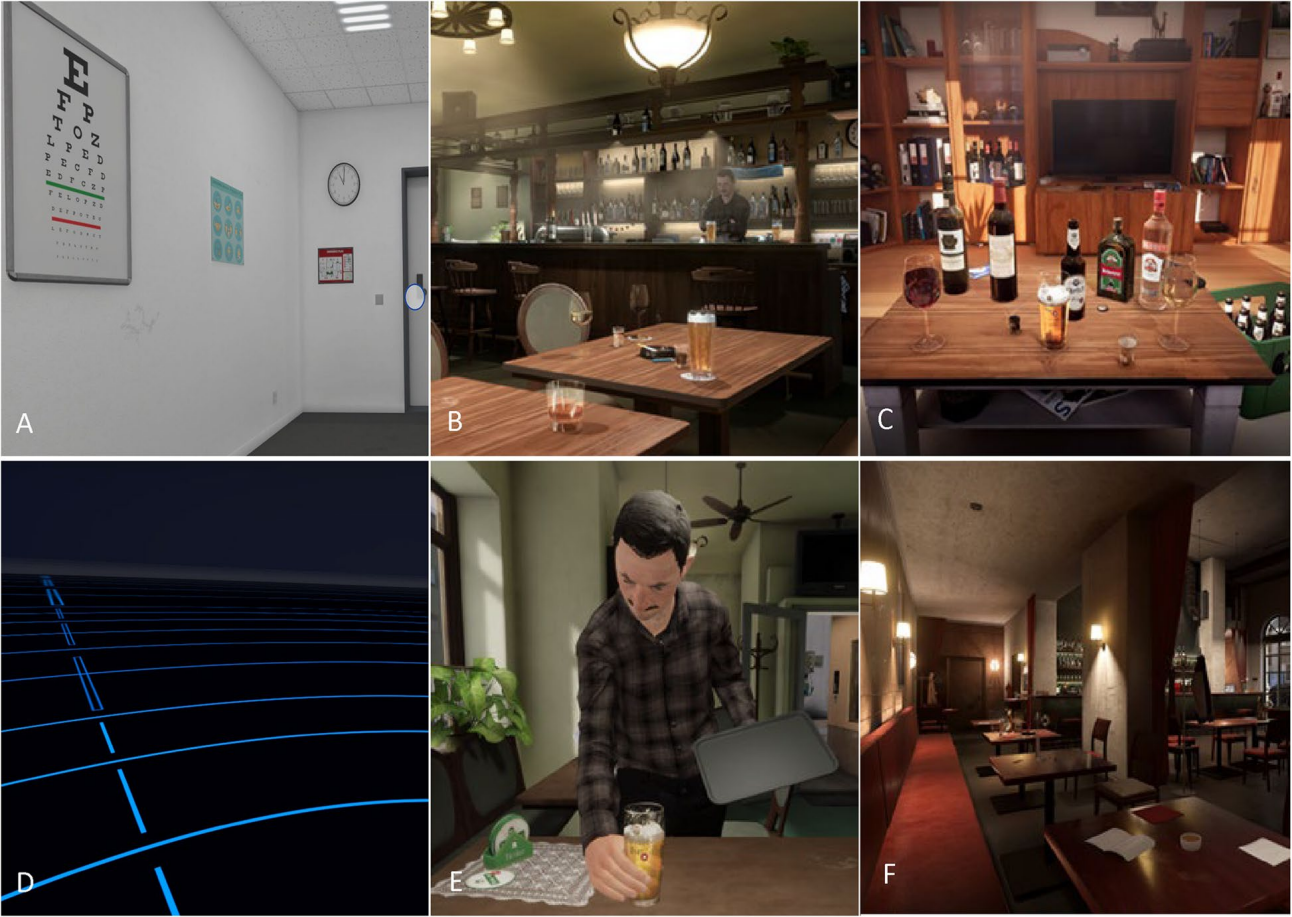
Virtual Reality scenarios of the CRAVE VR paradigm. **A**: VR acclimation room before starting the exposure session. **B**: Bar 1, a corner pub. **C**: Living room; patients choose one of the drinks depicted here before starting the VR exposure. **D**: Neutral VR scenario (baseline). **E**: Barkeeper in Bar 1, bringing a beer. **F**: Bar 2, an elegant wine bar

The necessary hardware used for the VR exposure include a VR head-mounted display (HTC VIVE Pro Eye) and a desktop PC based on SCHENKER XR Station with Intel Core i5-12,500. Patients will be asked to indicate their drink of choice (schnaps, red wine, white wine, beer or vodka) and choose between different possible bars for the VR exposure (see suppl. material for further details). According to patient’s preference regarding their drinks and bar specifications the VR scenarios will be individualized to these characteristics. Subsequently, all patients start with a VR acclimation session (approximately 1 but up to 5 min.) with unspecific context (clean, white waiting room; Fig. 2A) in order to accustom to the VR experience, headset and biosensors to avoid an arousal reaction during the experimental VR session. The exposure starts in a neutral environment (baseline): a “non-room” (black room with an indicated horizon and a bright grid for spatial orientation; Fig. 2D). Next, patients are confronted with the first of two high-risk scenarios in a randomized order (target situations): a) sitting on a couch in a living room, alone, with their pre-selected drink on a couch table before them (Fig. 2C), b) being in their bar of choice (Fig. 2B and F) surrounded by other people where the bar-keeper serves their pre-selected drink, puts it on the table in front of the patient and tells them to enjoy it (Fig. 2E). After the first risk situation, patients stop the VR exposure for up to 45 min allowing craving to decrease. Then, they enter the neutral environment (“non-room”) again before being exposed to the second risk situation (living room/bar). The duration of exposure to each VR situation (both baseline conditions, bar and living room) is 5 min, resulting into an overall VR exposure of 20 min.

#### Outcomes

Primary outcomes of the study are changes in craving levels measured by psychophysiological parameters (HR, HRV with special focus on HF-HRV, EDA, pupillometry) and subjective parameters (VAS) before, during and after VR-exposure to alcohol-associated cues compared to neutral cues.

Secondary outcomes are a) changes in subjective and objective craving levels compared between the two VR-scenarios bar vs. living room, b) the changes of subjective craving over the following 3 h after VR-exposure, c) measures of the sense of presence (scores of IPQ), d) measures of motion sickness assessed with the Fast Motion Sickness Scale.

#### Data management and monitoring

Clinical data and questionnaires will be collected using REDCap electronic data capture tools hosted at Charité—Universitätsmedizin Berlin [41, 42]. Data will be pseudonymized by unique identification codes for every patient entering the study. The reidentification list will be kept in a secure environment and only study responsible staff will have access. According to good scientific practice, the primary data are kept for ten years after publication. Data will be made available via the open science framework in a de-identified format and in the appendix of the final publication.

The highest possible data protection standards and secure data transmission protocols will be established following the EU General Data Protection Regulation and the Berlin Data Protection Act. The data will be stored on a designated server. All participants are entitled to inspect the data collected and/or to request the blocking or deletion of their data until deletion of the reidentification list.

#### Sample size

In previous studies on related topics, alcohol-associated cues compared to neutral cues show small to moderate effects on different psychophysiological parameters (0.2 < Cohen’s d < 0.39) and on subjective craving (Cohen’s d ~ 0.45) [6]. These previous studies used isolated “in vivo” stimuli. Due to a higher ecological validity of VR scenarios, we expect larger effects regarding the psychophysiological parameters (Cohen’s d = > 0.4). The sample size calculated in G-Power for computing ANOVA assuming an alpha error of 0.05 and a test power of α = 0.8 is *n* = 52. Assuming a drop-out rate of approximately 15%, 60 patients should be recruited to ensure sufficient statistical power.

#### Statistical methods

Statistical analyses are planned to be conducted using the software SPSS (SPSS Inc., Chicago, IL, United States). Growth-curve mixed models will be used to examine the effect of the three scenarios as within subject-condition (neutral, living room, bar) on physiological and subjective craving parameters. To address the change of subjective craving following the exposure, growth-curve mixed models will be used to examine the effect time (follow-up immediately, after 1, 2 and 3 h after VR exposure as within subject-factor with 4 levels) on craving levels as assessed by VAS.

#### Patient and public involvement

Patients were involved in study planning by stating appropriate stimuli in five anonymous clinical interviews on the ward for Treatment of Addiction Disorders in the Psychiatric University Hospital Charité at St. Hedwig-Hospital.

## Discussion

This is the first study examining change in several psychophysiological craving parameters in patients with AUD using VR exposure.

Results shall lead to a better understanding of the induction of craving in VR cue exposure as well as the link between subjective and physiological parameters. This could offer new diagnostic and therapeutic perspectives, e.g., providing a basis for future biofeedback training in VR exposure therapy. Since evidence on the effectiveness of CET in AUD is still limited [9, 10] and the role of habit learning in addictive disorders has been discussed (e.g. Hogarth et al. [43]), it would be valuable to discriminate subgroups with pronounced cue-reactivity who could potentially profit even more from this treatment. On an exploratory basis, the comparison between craving levels in the bar versus home situation will help to define contexts with pronounced craving, helping to better understand the role of social cues in inducing craving. As regards the direct benefit for patients, the VR experience in this study and future VR application can be an individually tailored tool to identify high-risk situations and practice coping skills.

This study is limited by a single-arm design: The study design does not allow a comparison of the elicited craving between patients and healthy controls. This would have changed the focus and research question of the study but would certainly be of interest for further studies in this field.

### Supplementary Information

**Additional file 1. **CRAVE VR paradigm.

## Data Availability

The datasets used and/or analysed during the current study are available from the corresponding author on reasonable request.

## Abbreviations

AUD: Alcohol use disorder
VR: Virtual reality
GDP: Gross domestic product
CET: Cue-exposure therapy
CBT: Cognitive behavioral therapy
HMD: Head-mounted display
SUD: Substance use disorder
AUQ: Alcohol Urge Questionnaire
VAS: Visual analogue scale
AUDIT: Alcohol Use Disorders Identification Test
OCDS: Obsessive Compulsive Drinking Scale
CAS-A: Craving Automated Scale for Alcohol
LDH: Lifetime Drinking History
IPQ: Igroup Presence Questionnaire
HR: Heart rate
HRV: Heart rate variability
EDA: Electrodermal activity
