# Supplementary material for: An explorative single-arm clinical study to assess craving in patients with alcohol use disorder using Virtual Reality exposure (CRAVE)—study protocol
Source: BMC Psychiatry. 2023 Nov 14;23:839. doi: 10.1186/s12888-023-05346-y (PMC10647047; doi:10.1186/s12888-023-05346-y)
Supplement: Supplementary file 1 — Additional file 1. CRAVE VR paradigm. [file 12888_2023_5346_MOESM1_ESM.docx]

**Supplementary Material**

*CRAVE VR paradigm*

Before the VR exposure patients indicate their preferred drink and choose between two different bars for the VR exposure: an elegant wine bar with warm light, red cushioned seating areas and a well-equipped wine counter or a rustical corner pub with a gaming machine and wooden chairs. Both bars are also visited by other people, alone or as couples, drinking their drink. Background sounds, such as soft music and bar noises are played over the head sounds.

All patients start with a VR acclimation room in a waiting room with an optometrist’s eye chart, a wall clock and an emergency plan hanging at the wall but without alcohol-related context. During this acclimation phase patients are asked to look at e.g. the watch or the eye-chart to assure that the eye-calibration was successful. Then, as soon as patients indicate that they are prepared to start, the baseline situation is faded in. This neutral VR scenario (see Fig. 2 D) is a black space with an indicated horizon and a bright grid for spatial orientation. After 5 minutes, patients enter the first risk situation: either the selected bar described above (wine bar or pub) or the living room. In both bars, the bar-keeper comes at their table, shortly after the start and brings their selected drink. He puts it on the table in front of the patient and wishes them to enjoy the drink (in German: “Sooo, lassen Sie es sich schmecken”).
The living room (see Fig. 2C) is a comfortable room with warm light, diverse interior design and multiple items like mobile phone, laptop, books lying around, providing a realistic atmosphere. On the table in front of the patient, they can find their selected drink. Through the headphones of the HMD they hear distant background sounds of the street.

After the first risk situation (5 minutes), patients stop the VR exposure for up to 45 min allowing craving to decrease. Then, the exposure continues with the second baseline room (5 minutes; again the described black room) being followed by the other risk situation (5 minutes; the selected bar or the living room).
